# Supplementary material for: Anti-Coronavirus Activity of Chitosan-Stabilized Liposomal Nanocarriers Loaded with Natural Extracts from Bulgarian Flora
Source: Life (Basel). 2024 Sep 19;14(9):1180. doi: 10.3390/life14091180 (PMC11605225; doi:10.3390/life14091180)
Supplement: Supplementary file 1 [file life-14-01180-s001.zip › life-3152607-supplementary.pdf]

Supplementary information

Figure S1. The electrokinetic potential of the liposomes loaded with a plant extract from *Sambucus nigra* (L_{SN}), *Potentilla reptans* (L_{PR}), *Allium sativum* (L_{AS}), *Aesculus hippocastanum* (L_{AH}), and as a function of the concentration of three different chitosans added to the dispersion: (●) CS-L, (○) CS-H, and (▲)

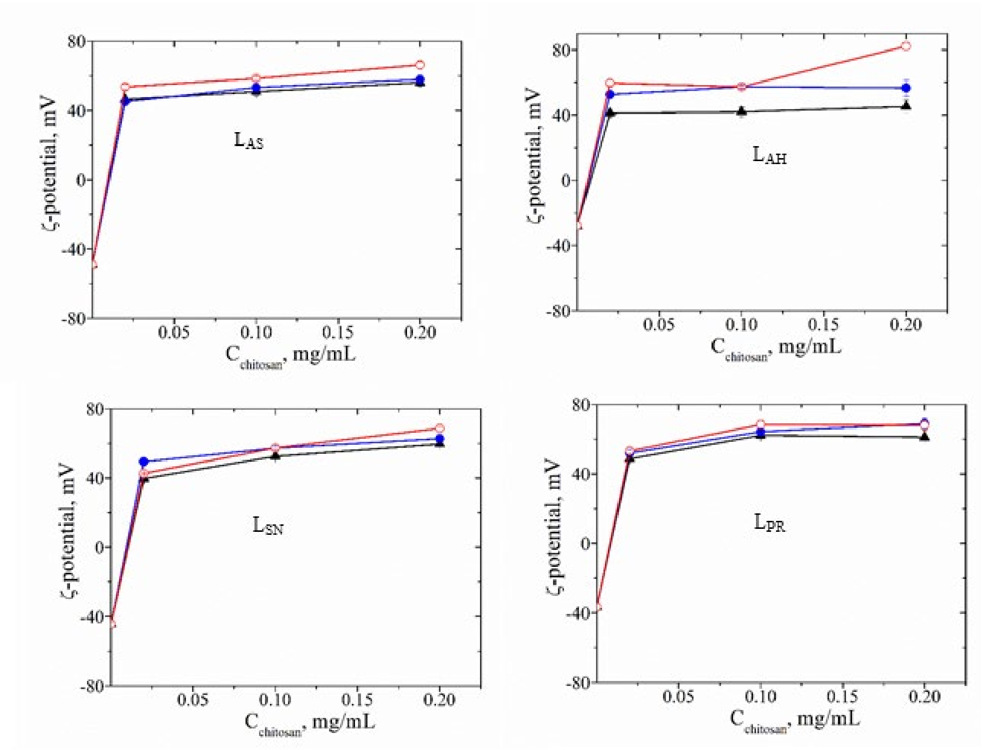

COS.
